# The Effects of Neuronal *Fyn* Knockdown in the Hippocampus in the Rat Kainate Model of Temporal Lobe Epilepsy

**DOI:** 10.3390/cells14100743

**Published:** 2025-05-19

**Authors:** Nikhil S. Rao, Marson Putra, Christina Meyer, Sirisha Parameswaran, Thimmasettappa Thippeswamy

**Affiliations:** Department of Biomedical Sciences, College of Veterinary Medicine, Iowa State University of Science and Technology, Ames, IA 50011, USAsirisha@iastate.edu (S.P.)

**Keywords:** *fyn* knockdown, Src family kinase, epilepsy, neurodegeneration, neuroinflammation

## Abstract

Previous studies have demonstrated neuronal and microglial Fyn, a Src family kinase (SFK), and how its interactions with tau contribute to epileptogenesis. Saracatinib, a Fyn/SFK inhibitor, modifies disease progression in rat kainate (KA) epilepsy models. In this study, we investigated neuronal-specific *fyn* knockdown effects on Fyn–tau signaling, neurodegeneration, and gliosis using a calcium/calmodulin-dependent protein kinase II (CaMKII)-promoter-driven adeno-associated viral vector (AAV9)-mediated *fyn*-shRNA injection in the rat hippocampus. Eight days following AAV administration, rats received repeated low-dose KA injections intraperitoneally to induce *status epilepticus* (SE). Both *fyn*-shRNA and control groups showed comparable SE severity, indicating inadequate neuronal *fyn* knockdown at this timepoint. Two weeks post *fyn*-shRNA injection, hippocampal Fyn significantly decreased, alongside reductions in NR2B, pNR2B^Y1472^, PSD95, and total tau. There was also a compensatory activation of SFK (pSFK^Y416^:Fyn) and tau hyperphosphorylation (AT8:total tau), negatively correlating with NeuN expression. Proximity ligation assay indicated unchanged Fyn–tau interactions, suggesting tau interactions with alternative SH3 domain proteins. Persistent neuronal loss, astrogliosis, and microgliosis suggested limited effectiveness of neuronal-specific *fyn* knockdown at this timepoint. An extended-duration *fyn* knockdown study, or using broad SFK inhibitors such as saracatinib or tau-SH3 blocking peptides, may effectively prevent SE-induced epileptogenesis.

## 1. Introduction

Epilepsy is the fourth most common neurological disorder and affects around 50 million people worldwide, with an estimated 3.4 million in the American population alone [1,2]. The current antiseizure medications are not effective in about a third of people with epilepsy [3,4,5]. Investigating alternate pathways of disease modification that target the early phase of epileptogenesis, in addition to the conventional neuronal ion-channel-targeted therapies, is an upcoming area of interest in preclinical studies [6,7,8,9]. Epileptogenesis is the process that leads to the development of spontaneous seizures by transforming the normal brain into an epileptic state, which is characterized by neuroinflammation, neurodegeneration, and oxidative stress [10,11,12].

We, and others, have demonstrated the role of Fyn kinase, a Src family tyrosine kinase (SFK), in neuroinflammation and epileptogenesis through the activation of microglia in Parkinson’s disease and in *status epilepticus* (SE)-induced rodent models of temporal lobe epilepsy [13,14]. In neurons, the role of Fyn and its interaction with tau in mediating hyperexcitability has been reported in Alzheimer’s disease [15]; moreover, very recently, we demonstrated a significant increase in Fyn–tau interactions in surgically resected temporal lobe from human epilepsy and rat models of temporal lobe epilepsy [16]. Fyn interacts with phosphorylated tau at Tyr^18^ in neurons and translocates the Fyn–tau complexes to the post-synaptic density of the synaptosomes and phosphorylates NR2B subunit of the NMDA receptor at Tyr^1472^, leading to prolonged activation of the receptor and excessive calcium influx [15,17]. Thus, selectively targeting neuronal Fyn presents an exciting opportunity for disease modification in epilepsy.

Treating with saracatinib, a broad-spectrum SFK inhibitor, significantly reduced nitrooxidative stress, neuroinflammation, neurodegeneration, epileptiform spikes (hyperexcitability), and spontaneous seizures in the rat kainate (KA) model of epilepsy [14,16]. These findings suggest that Fyn is a potential target for disease modification. Since Fyn–tau interaction causes hyperexcitability of neurons and neurodegeneration, we hypothesized that disengaging Fyn–tau interactions in neurons through *fyn* knockdown or the Fyn reduction approach will protect neurons and prevent neuroinflammation by modulating gliosis. To achieve this, we constructed a recombinant adeno-associated viral (AAV) vector to drive *fyn*-shRNA under a neuronal-specific promoter, calcium/calmodulin-dependent kinase II (CaMKII), and injected it into the rat hippocampus bilaterally at four sites to achieve maximum transduction. A week later, the animals were challenged with repeated low doses of KA to induce SE. A week after the induction of SE, we investigated the Fyn–tau interacting signaling molecules and the markers of neuroinflammation (gliosis) and neurodegeneration to determine whether selective hippocampal neuronal *fyn* knockdown modulates SE-induced epileptogenic markers and Fyn-associated signaling molecules.

## 2. Materials and Methods

### 2.1. Animals and Ethics Statement

In this study, we used young adult male Sprague Dawley rats aged 7–8 weeks procured from Charles River (Wilmington, MA, USA). The animals were single-housed and maintained at the Laboratory Animal Resources facility at Iowa State University in a controlled environment (19–23 °C, 12 h light: 12 h dark cycle), with unrestricted access to food and water. All experiments were conducted in compliance with the approved protocols (IACUC-21-110) of the Institutional Animal Care and Use Committee (IACUC). All animal studies were performed in accordance with the approved IACUC protocols of Iowa State University and ARRIVE (Animal Research: Reporting of In Vivo Experiments) guidelines. The animals were euthanized with 100 mg/kg pentobarbital sodium (i.p.), as per the American Veterinary Medical Association Guidelines for Euthanasia at the end of this study.

### 2.2. Recombinant Adeno-Associated Viral (rAAV) Vectors

The purified rAAV vectors were designed and synthesized by Vector Builder Inc. (Chicago, IL, USA). We used an Ultra-purified miR30-based scramble (SCR) shRNA control AAV9 vector (>10^13^ GC/mL, 10 × 100 uL) and an Ultra-purified custom miR30-based shRNA AAV9 vector (typical titer: >2 × 10^13^ GC/mL, minimum titer: >10^13^ GC/mL, 10 × 100 uL) for *fyn* knockdown. The technical specifications of the vectors are listed in Table 1, and the corresponding vector map is included in Figure S1. The AAV9 vector constructs were selected based on our preliminary studies that determined effective transduction using an eGFP reporter and robust *fyn* knockdown in the rat hippocampus.

### 2.3. Chemicals and Reagents

Kainic acid hydrate (Cayman Chemical, Ann Arbor, MI, USA) was dissolved in sterile water at 5 mg/mL. Diazepam and euthanasia solution (pentobarbital sodium and phenytoin sodium) were obtained from the Lloyd Veterinary Medical Center pharmacy at Iowa State University.

### 2.4. Experimental Design, Experimental Groups, and Kainate Exposure

**Figure 1:**
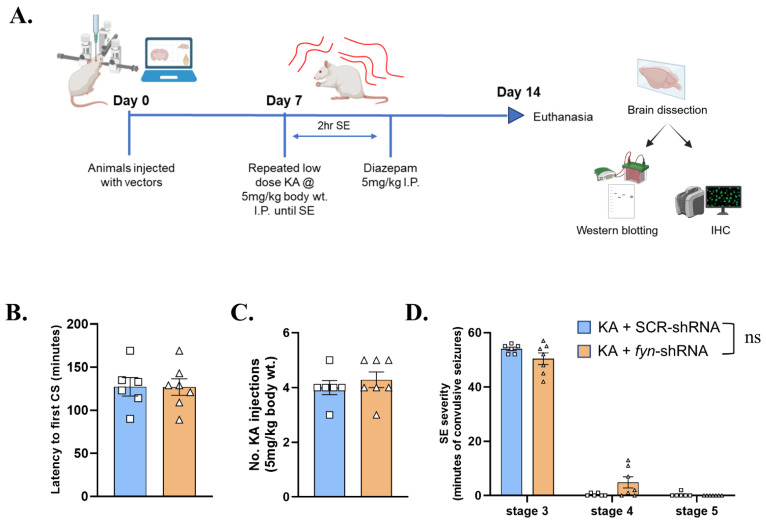
Experimental design and SE characteristics after KA-induced SE. (**A**) Experimental design. Created in BioRender https://BioRender.com/d74m332 (accessed on 15 May 2025). (**B**,**C**) There were no significant differences in the latency to the onset of convulsive seizures (CS) nor in the number of repeated low-dose KA injections required to induce SE in the *fyn* knockdown versus SCR-shRNA (scramble) groups. (**D**) After the onset of SE, there were no differences in SE severity scores between the groups. (**B**,**C**) Unpaired *t*-test; (**D**) Two-way ANOVA (Šídák’s multiple comparisons test). *n* = 6–7. Data represented as mean ± SEM.

The experimental design is illustrated in Figure 1A. The animals were randomized and grouped to receive either scramble shRNA vectors or *fyn* knockdown vectors. A separate set of rats, untreated with vectors, was used as the naïve control. The animals were stereotaxically injected with vectors and allowed to recover from surgery for a week before being challenged with KA. On day 8 post injection of vectors, repeated low doses of KA were administered intraperitoneally (5 mg/kg every 30 min) to induce *status epilepticus* (SE), as described previously [16,18]. Diazepam (5 mg/kg, i.p.) was administered two hours after the onset of the first convulsive seizure. One week later (day 8 post-SE), the animals were euthanized, and trans-cardiac perfusion was performed using ice-cold phosphate-buffered saline (PBS) for 10 min. The brains were dissected, and one hemisphere was placed in 4% paraformaldehyde for fixation and further histological processing, while the other half was flash-frozen in liquid nitrogen and stored at −80 °C for Western blot analysis.

### 2.5. In Vivo Vector Injections and Stereotaxic Surgery

**Figure 2:**
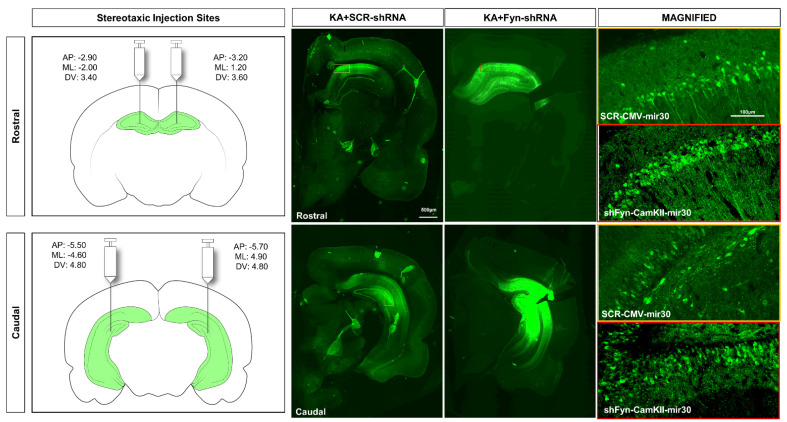
Stereotaxic injection coordinates. Rat brain schematics (not to scale) showing the sites for stereotaxic injection of AAV vectors in the hippocampus (highlighted green). Representative images of eGFP expression in the rostral and caudal hippocampus of the rat brains injected with SCR-CMV-mir30 (scramble) or *fyn*-shRNA-CamKII-mir30 (*fyn* knockdown) at 8 days post-SE.

The vectors were injected at four sites (two on each cerebral hemisphere) to achieve maximum transduction. Stereotaxic coordinates were derived from the online rat brain atlas (labs.gaidi.ca/rat-brain-atlas) to target rostral, middle, and caudal hippocampal regions. The injection sites were determined in the preliminary studies to ascertain the ideal sites for injections into the rostral to caudal hippocampi bilaterally. The coordinates for injections and representative images of the vector injections showing successful eGFP transduction at the targeted sites of the vectors in the hippocampus are presented in Figure 2. Stereotaxic injections were performed using a computer-guided robotic stereotaxic apparatus equipped with drill and microinjection (Stoelting Co., Wood Dale, IL, USA) and rat brain atlas integration for precise coordinate mapping and injection (Robostar software, version RCS8000). One µL of the ultra-purified vector (~10^10^ GC) was aspirated in a sterile Hamilton syringe (Stoelting Co., USA) and mounted on the microinjector drive of the stereotaxic robot. The animals were anesthetized with 3% isoflurane for induction, followed by 1.0–1.5% maintenance throughout the procedure. Buprenorphine hydrochloride (0.3 mg/kg, s.c.) and normal saline were administered immediately after the induction of anesthesia, and artificial tears ointment was applied to the eyes. The surgical site was prepared and sterilized with chlorhexidine and isopropyl alcohol. A mid-sagittal incision was made on the skin over the skull, and the skin was reflected and secured using hemostats. The underlying tissues were resected to expose the surface of the skull. Holes were drilled through the skull at the injection sites using precise coordinates set in the Robostar software (Figure 2). The injection arm of the stereotaxic robot loaded with the Hamilton syringe was then lowered into the hole to reach the target coordinate in the hippocampus. Slow injection of vectors was carried out at a rate of 1 uL/min. The surgical site was closed using 9 mm wound clips, and topical triple antibiotic ointment (Vetropolycin) was applied. Baytril (enrofloxacin, 5 mg/kg, s.c.) and 1 mL of 5% dextrose normal saline were administered postoperatively. Critical care diet and dextrose normal saline injections were continued for 3 days postoperatively to aid recovery.

### 2.6. SE Induction and Initial SE Severity Scoring

SE severity scoring was performed as previously described in our publications based on a modified Racine scale for the KA model [16,18,19]. Seizure stage scoring was based on direct observation of the animals’ behavior by trained experimenters. The total number of minutes that an animal spent in convulsive seizure stages (Stage 3 to Stage 5) from the first onset of convulsive seizure to diazepam injection was summed up to derive the SE severity score. The stages of seizures, as previously described, are as follows: Stage 1—is characterized by freezing and immobility; Stage 2—wet dog shakes, facial automatism, and head nodding; Stage 3—rearing with forelimb clonus and/or Staub tail; Stage 4—rearing with forelimb clonus and falling; and Stage 5—loss of righting reflex and/or violent jumping or lateral recumbent with limbs clonus.

### 2.7. Euthanasia, Tissue Processing, Immunohistochemistry (IHC), Imaging, and Cell Quantification

The animals were euthanized on the 8th day post-SE with an intraperitoneal injection of euthanasia solution (pentobarbital sodium and phenytoin sodium). After trans-cardiac perfusion and brain dissection, one half of the cerebral hemisphere was placed in 4% paraformaldehyde solution for 24 h at 4 °C for fixation, followed by cryoprotection with 25% sucrose in 0.1 M PBS for 72 h in the fridge. Embedding of the tissue was carried out in porcine gelatin Type A (Sigma Aldrich, Saint Louis, MO, United States), followed by flash freezing in supercooled isopentane with liquid nitrogen and stored at −80 °C. Cryosectioning at 16μm thickness was carried out using a cryostat (Leica Microsystems, Wetzlar, Germany) and mounted on precleaned gelatin-coated slides. Starting from the rostral hippocampus caudally, five coronal sections were mounted on each slide so that the distance between successive brain sections on each slide was approximately 480 μm. The slides containing sections were archived and stored at −20 °C until further processing for immunostaining. The brains were processed for IHC as previously described [16,18]. For IHC, antigen retrieval was carried out by heating the slides containing the brain sections in citric acid buffer (10 mM citric acid, 0.05% tween 20, pH 6.0) at 95 °C for 20 min. The slides were washed with PBS, followed by blocking with 10% donkey serum for an hour at room temperature. Primary antibody incubation was carried out overnight at 4 °C. The next day, the slides were washed with PBS and incubated for an hour with a secondary antibody conjugated to a fluorophore. The slides were washed and applied with Vectashield^®^ anti-fade mounting medium. The antibodies used for IHC in this study were as follows: Goat anti-IBA1 (1:200, Abcam, Waltham, MA, USA), rabbit anti-NeuN (1:200, EMD Millipore, Burlington, MA, USA), mouse anti-GFAP (1:200, Sigma Aldrich), Rhodamine Red X donkey anti-rabbit (1:200, Jackson ImmunoResearch, West Grove, PA, USA), biotinylated donkey anti-goat (1:200, Jackson ImmunoResearch), and AlexaFluor 488 donkey anti-mouse (1:200). Cy3-conjugated streptavidin (1:100, Jackson ImmunoResearch) was used to detect the biotinylated antibody. Image acquisition was carried out using a Leica DMi8 (Leica, USA) inverted fluorescent microscope or Keyence BZ-0023 (Keyence, Osaka, Japan). For IBA1 and GFAP cell counts, images from the hippocampal regions—dentate gyrus (DG), CA3, CA1, and subiculum (SUB)—were acquired at 20X magnification, quantified, and averaged across a minimum of 2 sections per animal. Cell quantification was performed using automated processes, as described previously, using ImageJ V2.9.0 and CellProfiler V4.2.8 [18].

### 2.8. Proximity Ligation Assay

In situ proximity ligation assay (PLA) was performed on brain sections as previously described [16,20]. We used the Duolink In Situ PLA kit (Sigma-Aldrich, USA) for the assay. The brain sections were subjected to antigen retrieval followed by blocking with the blocking buffer using the manufacturer’s recommended protocol. The sections were incubated with primary antibodies against Fyn (Sigma-Aldrich, HPA023887) and tau (DA9, RRID: AB_2716723) to detect interactions, while MAP2 (Invitrogen, Waltham, MA, USA, PA1-10005) was used to visualize the Fyn–tau complexes within dendrites. The remaining steps were carried out as per the manufacturer’s protocol. Appropriate negative controls comprising sections without primary antibodies were run in tandem with the samples.

### 2.9. Western Blotting

Western blotting was carried out as per previously described protocols [16,21]. Whole hippocampal lysates were prepared in RIPA buffer (ThermoFisher, Waltham, MA, USA) containing 1% protease and phosphatase inhibitors. The lysates were resolved on 8–12% SDS-PAGE gels and transferred onto nitrocellulose membranes (Bio-Rad, Hercules, CA, USA). The membranes were blocked using TBS-based fluorescent blocking buffer (Li-Cor, Lincoln, NE, USA). Primary antibody followed by secondary antibody incubations and washing were performed. Details of the antibodies used for Western blotting are listed in Supplementary Table S1. The membranes were scanned using the Odyssey 9120 IR scanner (Li-Cor, USA) to detect the target proteins. Exported images of the blots were quantified using the densitometry analysis in ImageJ (NIH) and normalized to beta-actin to calculate relative fold changes versus controls. To study the phosphorylation states of certain proteins, the ratio of their phosphorylated form to native forms was computed. Full blot images are presented in Figure S4.

### 2.10. Statistical Analysis

We used GraphPad Prism v10 for generating graphs and statistical analyses. The datasets were first tested for normality using the Shapiro–Wilk test. For data with normal distribution, we used the unpaired *t*-test for two group comparisons and ordinary one-way ANOVA with post hoc correction for multiple group comparisons. For data with non-normal distribution, we used the Kruskal–Wallis test with post hoc correction for multiple group comparisons. Details of the exact statistical tests used are described in the respective figure legends. Bar graphs were plotted as mean ± standard error of the mean (SEM) with individual datapoints.

## 3. Results

### 3.1. Initial SE Severity

Repeated low doses of KA were administered to determine the impact of *fyn* knockdown on latency to the onset of SE and its severity. There were no significant differences in the latency to the onset of convulsive seizures (CS) (Figure 1B) or the number of repeated low doses of KA required to induce SE between the scramble-shRNA-treated and the *fyn*-shRNA-treated groups (Figure 1B). The initial SE severity score between the scramble-shRNA (control) versus the fyn-shRNA-treated rats also did not differ significantly (Figure 1D).

### 3.2. Fyn Knockdown Reduced Fyn but Increased pSFK^Y416^ to Fyn Ratio in the Hippocampus at 8 Days Post-SE

**Figure 3:**
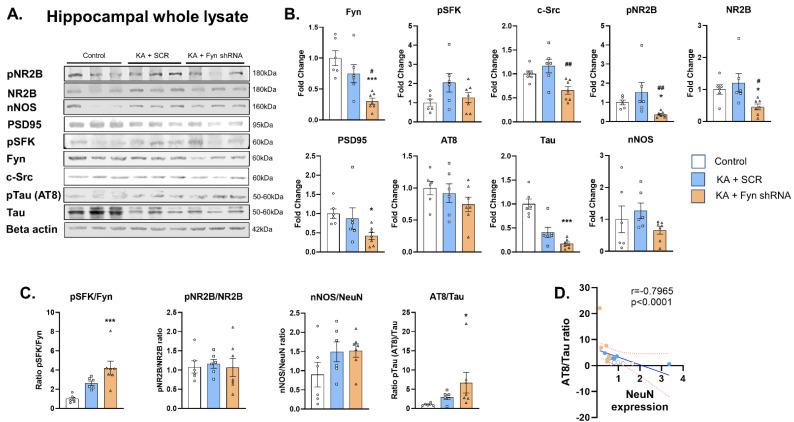
Western blot analysis of the whole hippocampal lysates probed for Fyn–tau interacting molecules. (**A**) Representative Western blots of the hippocampal whole cell lysates probed for pNR2B, NR2B, nNOS, PSD95, pSFK, Fyn, c-Src, pTau (AT8), and total tau and beta-actin. (**B**) Densitometric analysis of the Western blots. (**C**) Ratios of relative proteins of the markers probed in the Western blots. (**D**) There was a significant negative correlation between the hyperphosphorylated tau (AT8) and NeuN positive neurons. (**B**,**C**) One-way ANOVA (Tukey’s multiple comparisons test) or Kruskal–Wallis test (Dunn’s multiple comparisons test), n = 6–7. Data represented as mean ± SEM. (**D**) Spearman correlation with simple linear regression. The dotted lines represent the 95% confidence interval of the mean, n = 6–7. * *p* < 0.05, *** *p* < 0.001 vs. control; # *p* < 0.05, ## *p* < 0.01 vs. KA + SCR. Data represented as mean ± SEM.

To validate the hippocampal-targeted *fyn* knockdown, we performed Western blotting of the whole hippocampal lysates for Fyn levels from *fyn*-shRNA and control groups. As anticipated, we found a significant reduction in total Fyn in the knockdown group versus the scramble-treated group and the naïve control (Figure 3A,B). The levels of pSFK^Y416^ did not differ significantly between the treatment groups despite there being significant differences in the levels of Fyn (Figure 3A,B). However, the ratio of pSFK^Y416^ to total Fyn was significantly increased in the *fyn* knockdown group versus the controls, indicating a compensatory activation of other SFK members (Figure 3C). To further confirm hippocampal-specific *fyn* knockdown, the extra-hippocampal region, the piriform cortex, was probed for Fyn. The Western blotting analysis of this non-targeted brain region did not show significant differences in Fyn levels between the groups (Figure S2).

### 3.3. The Effects of Fyn Knockdown on Fyn Interacting Molecules: Src, pNR2B, NR2B, PSD95, pTau (AT8), Total Tau, and nNOS Expression in the Hippocampus

**Figure 4:**
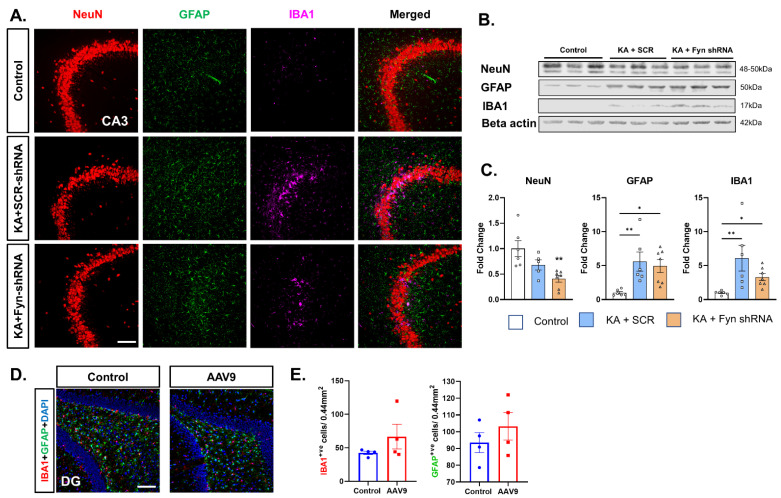
Gliosis and neurodegeneration in the hippocampus. (**A**) Representative images of the CA3 region of the hippocampus of rats from different treatment groups immunostained for NeuN (neurons), GFAP (astrocytes), and IBA1 (microglia). Scalebar 100 μm. (**B**) Representative Western blots of the whole hippocampal lysates of rats from different treatment groups at 8 days post-SE. (**C**) *Fyn* knockdown, compared to the control, exacerbated KA-induced neurodegeneration by reducing NeuN expression. *Fyn* knockdown did not mitigate KA-induced astrogliosis (GFAP expression) and microgliosis (IBA1 expression) in the hippocampus. (**D**) Representative images of the DG region of the hippocampus of control and AAV9-vector-injected rats (without KA) immunostained for GFAP, IBA1, and DAPI. Scalebar 100 μm. (**E**) Injection of AAV9 vectors alone did not cause significant increases in gliosis compared to the control. (**C**) One-way ANOVA (Tukey’s multiple comparisons test), n = 6–7, * *p* < 0.05, ** *p* < 0.01 vs. control.; (**E**) Unpaired *t*-test, n = 4 per group. Data represented as mean ± SEM.

Western blot analyses of the hippocampus revealed a significant reduction in pNR2B^Y1472^ and total NR2B, PSD95, and total tau in the *fyn* knockdown animals (Figure 3A,B). The significant reduction in the levels of these proteins of neuronal origin suggests neuronal loss in the *fyn* knockdown group. There was, indeed, a significant reduction in neurons in the hippocampus in response to KA-induced SE (Figure 4B,C). However, there was no difference in the ratio of pNR2B/NR2B between the groups (Figure 3C). There was also no significant difference in the levels of pTau (AT8) and nNOS between the groups (Figure 3A,B). Since we observed significant reductions in the total tau and NeuN, we further examined the ratio of pTau (AT8)/total tau, which was significantly increased in the *fyn* knockdown group versus that of the controls (Figure 3C). We also found a strong negative correlation between the phosphorylation of tau (AT8) and NeuN expression, suggesting that hyperphosphorylated tau may be associated with neuronal loss in the *fyn* knockdown group post-SE (Figure 3D). There was an increase in the ratio of nNOS/NeuN in both of the KA-treated groups versus that of the control, but the differences were not statistically significant (Figure 3C).

### 3.4. The Effects of Fyn Knockdown on Fyn–Tau Interactions

**Figure 5:**
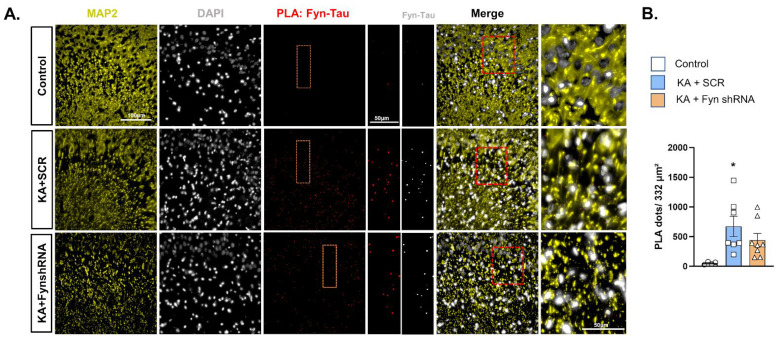
Fyn–tau interactions at 8 days post-SE. (**A**) Representative PLA images of Fyn–tau interactions in the CA3 region of the hippocampus of KA-treated rats. (**B**) Fyn knockdown did not significantly reduce Fyn-tau interactions versus the scramble-treated rats.(**B**) Ordinary one-way ANOVA (Tukey’s multiple comparisons test), n = 4–7. * *p* < 0.05 vs. control. Data represented as mean ± SEM.

To examine the effects of KA-induced SE in *fyn* knockdown animals on Fyn–tau interactions post-SE in the hippocampus, we used the PLA to visualize their interactions in situ. Representative images of the PLA for Fyn–tau interactions co-stained with dendritic marker MAP2 in the hippocampal CA3 region of the brain are presented in Figure 5A. As expected, we observed a significant increase in Fyn–tau interaction (PLA signals) in the scramble-vector-treated rats exposed to KA versus that observed in the naïve control. However, despite a significant reduction in Fyn levels in the *fyn* knockdown group versus the scramble-shRNA group, there was no significant reduction in Fyn–tau complexes in the *fyn* knockdown group (Figure 5B).

### 3.5. Hippocampal Neuronal Fyn Knockdown Did Not Prevent Neuronal Loss After SE and Also Had No Effect on SE-Induced Gliosis

From a translational perspective, we examined whether hippocampal neuronal *fyn* knockdown post-SE dampens the markers of epileptogenesis, such as neurodegeneration (NeuN expression), astrogliosis (GFAP expression), and microgliosis (IBA1 expression), in the hippocampus. Representative images of the hippocampal CA3 region of the brain immunostained for NeuN, GFAP, and IBA1 are presented in Figure 4A. Western blotting of the whole hippocampal lysates were probed for the markers of neurodegeneration and gliosis (Figure 4B). KA-induced SE led to a significant loss of neurons (NeuN) in the hippocampus of the *fyn* knockdown group versus that of the controls (Figure 4C). KA-induced SE caused a significant increase in astrogliosis (GFAP) and microgliosis (IBA1) in both scramble-shRNA- and *fyn*-shRNA-vector-treated groups versus the naïve control (Figure 4C). The intrahippocampal vector injections alone, without exposure to KA, did not significantly induce microgliosis or astrogliosis (Figure 4D,E).

## 4. Discussion

The goal of this study was to selectively reduce hippocampal neuronal Fyn tyrosine kinase, which is implicated in mediating neuronal hyperexcitability through its interactions with tau and phosphorylation of the post-synaptic NMDA subunit NR2B at Tyr^1472^ post-insult [15,16]. We hypothesized that reducing Fyn during post-SE in the hippocampal neurons, the seizurogenic foci in the KA model, would reduce neuronal hyperexcitability due to reduced Fyn interactions with the other signaling molecules and dampen the markers of epileptogenesis. The premise for this hypothesis was based on our published studies in the *fyn* knockout mouse KA and pentylenetetrazole (PTZ) models, and from the rat KA model treated with saracatinib, a Src/Fyn tyrosine kinase inhibitor, post-SE. In both mouse models, as expected, the latency to the onset of the first convulsive seizure and the overall SE severity were significantly reduced in *fyn* knockout mice compared to their respective controls [14,20]. In those mouse models, the real impact of the complete absence of Fyn on epileptogenic markers could not be ascertained due to the compromised initial SE severity. Nevertheless, the absence of Fyn reduced neuronal hyperexcitability in those mouse models. To further test whether inhibiting Fyn/SFKs after the induction of SE will modulate both hyperexcitability and the epileptogenic markers, we treated the rats with saracatinib after the induction of SE with repeated low-dose KA. In that model, the epileptogenesis was measured with real-time continuous video-EEG for several months [22]. In the saracatinib-treated rats, there was a significant reduction in spontaneously recurring seizures and epileptiform discharges, as well as the markers of epileptogenesis [16,22]. Though these observations are translational and clinically relevant, from mechanistic perspectives, we were interested in investigating whether selective neuronal Fyn reduction in the hippocampus alone would be sufficient to dampen the early markers of epileptogenesis, i.e., SE-induced neurodegeneration and gliosis.

AAV vectors have been highly effective in transducing the gene of interest in preclinical studies and clinical gene therapy trials due to their broad tropism, ease of synthesis, low immunogenicity, and non-pathogenicity [23]. AAV vectors are endocytosed upon attaching to the cell surface and transported to the nucleus, where the transgene is released to control the target gene expression [24]. Depending on the cell-specific promoters used to drive the transgene, the gene expression occurs in those cells. Initially, we tried CMV-promoter-driven *fyn*-shRNA (AAV) vectors to achieve neuronal Fyn reduction in primary mixed hippocampal neuron–glia cultures, but it was ineffective (Figure S5). We also tested several serotypes in vivo by stereotaxic injection into the hippocampus to select the most appropriate serotype (AAV2, AAV9, and AAVrh10) suitable for neurons. We found that miR30-based AAV9 with a neuronal-specific CaMKII-promoter-driven *fyn*-shRNA was most suited to target neuronal Fyn rather than the CMV promoter, as evident from extensive eGFP expression in the hippocampal pyramidal neurons but not in the glia (Figures S5 and S6). Since AAV9 gave a robust eGFP expression in the neurons, with little to no expression in the glia, the use of miR-30 with the CaMKII promoter was feasible to target the hippocampal neurons. There was eGFP expression in some of the extrahippocampal neurons, such as in the piriform cortex and the entorhinal cortex, due to the anterograde transport of vectors from the injection site in the hippocampus. However, there was no significant reduction in Fyn in the non-targeted brain region (Figure S2).

The introduction of AAV vectors into the brain could evoke localized gliosis of varying degrees. The extent of localized gliosis depends on the AAV serotypes, the degree of purification of vectors (ultra-purified vectors are low immunogenic), the viral particle load and volume injected, the targeted transgene itself, the surgical technique used (such as the size of the burr used to drill holes through the skull and avoiding the course of major blood vessels on the surface of the brain during injection), and the age of the animals used in the experiment [25,26,27,28,29]. Considering these factors, we tested whether vector injection alone, without KA challenge, would induce gliosis in our model. There were no significant differences between the naïve control and the vector-injected group, suggesting the gliosis observed in this study was due to the effect of SE induced by KA, but not due to the vectors per se (Figure 4D,E). We used ultra-purified AAV9 vectors that were least immunogenic, injected at four sites in low volumes, and targeted neuronal *fyn* to dampen neuronal hyperexcitability, thereby minimizing glial activation through neuron–glial communication; moreover, our expertise in surgical procedure minimized tissue damage, which may have prevented vector-induced gliosis in this study.

Based on the pilot studies, we chose day-8 post-vector injection for the KA challenge to achieve the required initial SE severity in both *fyn*-shRNA and control vector-injected groups (Figure 2). A further delayed KA challenge would have compromised the initial SE severity in the *fyn*-shRNA group due to a reduction in Fyn levels in the hippocampal neurons and may have confounded the markers of epileptogenesis, as in the *fyn* knockout mouse models [14,20]. Therefore, our chosen timing of the KA challenge was appropriate. However, despite the reduction in Fyn by day 8 post-SE, i.e., by two weeks post vector injections, we did not observe a reduction in epileptogenic markers, such as neuronal loss, microgliosis, and astrogliosis. There was neuronal loss in the hippocampus of the *fyn* knockdown group versus the controls, as indicated by the loss of NeuN expression and the corresponding decrease in the expression of PSD95, NR2B, and total tau. We also found a significant increase in hyperphosphorylated tau (AT8) in the *fyn* knockdown group (Figure 3C,D). Hyperphosphorylated tau promotes neurodegeneration [16,30,31]. These findings were completely unexpected and contradict our initial hypothesis that selective neuronal Fyn reduction dampens neuronal hyperexcitability and the markers of early epileptogenesis (gliosis and neurodegeneration). It is likely that the magnitude of Fyn reduction, despite bilateral injections at four sites, was not enough at the time point tested. In the future, a long-term study with video-EEG may be required to detect subtle changes in neuronal hyperexcitability. Alternatively, selectively inhibiting Fyn in neurons alone might not be sufficient to target epileptogenesis, considering the role of Fyn in microglia. Fyn has been implicated in neuroinflammation, mediated by PKC-delta in microglia in neurodegenerative disorders, including epilepsy [13,14,32]. However, the literature review did not reveal any studies that have attempted inhibiting Fyn selectively in hippocampal neurons in a model of epilepsy. Despite the limitations of this study, the findings demonstrate that selectively targeting hippocampal neuronal Fyn alone may not be beneficial considering the compensatory role of other SFKs such as c-Abl (Abelson kinase) and microglial Fyn in epileptogenesis [14,33,34].

The SFKs are a group of non-receptor tyrosine kinases and comprise nine member kinases, namely Fyn, Src, Yes, Lyn, Lck, Blk, Kck, Fgr, and Yrk [35]. In this study, selective *fyn* knockdown in the hippocampal neurons upregulated the phosphorylated SFK (pSFK^Y416^). The phosphorylation of SFKs at Y^416^ is not unique to Fyn, but rather conserved across all members of the SFKs, and also the autophosphorylation site of these kinases [35]. Therefore, the increased ratio of pSFK/Fyn in the *fyn* knockdown group may represent the activation of the residual *Fyn* or a compensatory increase in the activated form of other SFK members in neurons. In this study, we also tested the altered levels of c-Src, which were also significantly reduced in the *fyn* knockdown rats versus the scramble group in the KA-treated rats, which implies the role of other members of the SFKs other than Fyn [35,36]. It is also well-established that the other members of the SFKs can interact with tau through their SH3 domains to mediate physiological or pathological function based on the signal/stimulus [36,37]. Moreover, it is the tau phosphorylation at Y^18^ that mediates NMDA-receptor-mediated excitotoxicity and not necessarily the Fyn–tau interactions per se [38].

In the current study, there was a significant reduction in Fyn in the hippocampus but not complete ablation throughout the brain as seen in the mouse knockout models. There was a significant increase in the ratio of pSFK/Fyn in the *fyn* knockdown group, implying that the residual Fyn may have phosphorylated, too. The findings from in situ PLA strongly support this possibility, considering there was no significant reduction in PLA complexes in the *fyn* knockdown versus the scramble groups. The neuroinflammatory phenotype in the *fyn* knockdown group may have also resulted from the microglial Fyn-PKCδ activation [13,14]. These findings suggest that selective hippocampal neuronal Fyn reduction alone neither reduces seizure severity in response to seizurogenic agents nor dampens neuroinflammation post-SE. Therefore, global conditional knockdown of Fyn post-SE for a long-term or broad-spectrum SFK inhibitor, such as saracatinib, could be therapeutically beneficial to modify epileptogenesis [8,16,18,22].

There were no significant differences in the initial SE severity scores between the scramble-shRNA and *fyn* knockdown group in response to KA, nor were there any differences in the number of repeated low-dose KA injections (i.e., latency) required to induce SE in this study. These results differ from our previous study with the *fyn* knockout mouse PTZ model, where a 30% reduction in the SE severity versus the wild type was observed [20]. In another *fyn* knockout mouse KA study, we also observed reduced SE severity [39]. However, in the latter *fyn* knockout mouse KA study, mortality was high due to a single high dose of KA (25 mg/kg, i.p.) in contrast to the current rat KA study with repeated low doses of KA. It is also likely that hydrocephalus is an important phenotype in *fyn* knockout mice, implicating its critical physiological role in development [40] and their response to KA or PTZ [20,39]. The results from *fyn* knockdown in hippocampal neurons in the rat KA model in this study suggest that Fyn in other regions of the brain, or in the microglia or other SFKs, may have promoted KA-induced SE and epileptogenic markers [14,22]. The Western blot analysis of Fyn levels in the extrahippocampal brain region, the piriform cortex, did not show a reduction in Fyn (Figure S2), suggesting that the *fyn* knockdown was specific to hippocampal neurons.

Despite the attempt to target the hippocampal neurons bilaterally and at both rostral and caudal injections of the most suitable vectors, the magnitude of Fyn reduction observed during the early epileptogenic phase post-SE seems insufficient to dampen the epileptogenic markers. A long-term study with telemetry device implantation for continuous video-EEG acquisition could reveal if there would be a further delay and significantly greater reduction in Fyn later. The role of other SFKs, such as c-Abl, should also be considered in future studies, since the literature suggests its role in ictogenesis and epilepsy [33,34,41]. In addition, the outcome of this study also reveals that selective neuronal targeting may not be sufficient to dampen epileptogenesis, and microglial Fyn also contributes to pathogenesis during post-SE [14,22]. However, the rationale for targeting neuronal Fyn was that, by dampening neuronal hyperexcitability, we could also prevent microglial proliferation, since Fyn plays a role in cell proliferation and migration/invasiveness [42,43,44]. Targeting microglial Fyn using viral-vector-mediated gene delivery is technically challenging after inducing SE. In our preliminary studies, we tested the generic CMV promoter sequence inserted into AAV vectors and found that microglia were not successfully transduced, and Fyn reduction was not significant (Figures S3 and S5). However, the emerging novel approaches for selective microglial targeting can be useful for future studies [45,46,47]. Another limitation of the present study is the use of only male rats. Previously, we reported the early epileptogenic effects of KA-induced SE in a mixed-sex cohort of adult rats, and we did not observe any sex differences or observe a significant sex interaction in response to treatment with a pharmacological SFK inhibitor, saracatinib [18]. Therefore, we did not anticipate that there would be a significant sex difference in response to *fyn* knockdown in this study.

The results from this study targeting a single gene commensurate with results from another AAV-mediated gene (*nNOS*) knockdown study in the rat model of traumatic brain injury (TBI), which concluded that suppressing the activity of a single gene may not alter the functional outcome of the disease [48]. We recently demonstrated Fyn–tau interactions as a key mediator of hyperexcitability and a potential therapeutic target in temporal lobe epilepsy treatment [16]. We have also demonstrated that pharmacologically inhibiting SFKs using saracatinib reduces Fyn–tau interactions and modifies the progression of KA-induced epileptogenesis [16]. Saracatinib is a broad-spectrum SFK inhibitor. Another strategy is to selectively target Fyn–tau interactions using a blocking peptide. A novel tau-SH3 blocking peptide was tested in an in vitro Alzheimer’s disease model and has been shown to ameliorate amyloid-beta toxicity in neurons without affecting the activity of Fyn kinase [49]. Thus, inhibiting the Fyn–tau interactions by blocking the proline-rich domain of tau where Fyn and other SFKs associate with tau to cause its phosphorylation at Y^18^, or treating with a pharmacological broad-spectrum SFK inhibitor, such as saracatinib, could potentially modify epileptogenesis [22].

## 5. Conclusions

This study investigated the effects of selective *fyn* knockdown in the hippocampal neurons prior to the induction of SE using repeated low-dose KA in the rat model. *Fyn* knockdown in the hippocampal neurons alone did not prevent SE-induced neuronal loss, astrogliosis, or microgliosis at 8 days post-SE (2 weeks post vector injections). Despite the limited outcome, we demonstrated the optimum timing of vector injections for a gene knockdown of interest without compromising the initial SE severity, which is critical for epileptogenesis, and demonstrated at 2-weeks post-injection; the Fyn levels were significantly downregulated at the targeted site. Although we could not dampen the epileptogenic markers post-SE, which could be due to an unexpected compensatory increase in other phosphorylated SFKs in the *fyn* knockdown group at 8 days post-SE or the fact that the magnitude of Fyn reduction was not enough in the short-term study. From a mechanistic perspective, a further long-term study with continuous video-EEG acquisition could reveal the long-term effects of *fyn* knockdown on neuronal hyperexcitability and epileptogenesis. From a translational perspective, the results of this short-term study indicate that *fyn* knockdown, in isolation, may not be beneficial. Instead, pharmacological broad-spectrum SFK inhibitors, such as saracatinib, or blocking the Fyn–tau interactions using a tau-SH3 blocking peptide, may be relevant.

## Figures and Tables

**Table 1:** Details of vectors used in this study.

	Scramble	Fyn Knockdown
Vector ID	VB010000-9397wgw	VB220605-1062nve
Vector Name	pAAV[miR30]-CMV>EGFP:Scramble_miR30-shRNA:WPRE	pAAV[2miR30]- Camk2a(short)>EGFP:{rFyn[shRNA#3]}: {rFyn[shRNA#2]}:WPRE
Vector Type	Mammalian miR30-shRNA Knockdown AAV Vector	Mammalian Dual miR30-shRNA Knockdown AAV Vector
Inserted Promoter	CMV	Camk2a(short)
Inserted shRNA	Scramble_miR30-shRNA	{rFyn[shRNA#3]}, {rFyn[shRNA#2]}
Target Sequence	ACCTAAGGTTAAGTCGCCCTCG	GTGAAGGACGGGCTCTGAAATT, CTGTGTGCAATGTAAGGATAAA

## Data Availability

The original contributions presented in this study are included in this article/Supplementary Material, and further inquiries can be directed to the corresponding author.

## Supplementary Materials

The following supporting information can be downloaded at: https://www.mdpi.com/article/10.3390/cells14100743/s1, Figure S1. Vector maps of the recombinant AAV vectors used in this study; Figure S2. Fyn expression in the piriform cortex; Figure S3. Preliminary data for testing AAV vector constructs; Figure S4. Whole membrane scans of the Western blots of Figure 3 and Figure 4; Figure S5. Preliminary in vitro studies in primary neuro-glial cultures showed no reduction in Fyn expression in any of the shRNA-treated groups.; Figure S6. (A) Preliminary studies for AAV serotype testing to select effective serotypes for neuronal shRNA transduction. (B) AAV9 serotype yielded the most effective transduction of eGFP expression in hippocampal pyramidal neurons without any expression in glial cells. (C) Discrete expression of eGFP in the pyramidal neurons that also expressed CaMKII.

## Abbreviations

The following abbreviations are used in this manuscript:

SFK	Src family kinase
KA	Kainate
SE	*Status epilepticus*
CaMKII	Calcium/calmodulin-dependent protein kinase II
AAV	Adeno-associated viral vector
PLA	Proximity ligation assay
NMDA	N-methyl-D-aspartate
NR2B	NMDA receptor subunit 2B
PSD95	Post-synaptic density protein 95
pSFK^Y416^	Phosphorylated Src family kinase at tyrosine 416
pNR2B^Y1472^	Phosphorylated NR2B at tyrosine 1472
NeuN	Neuronal Nuclear Protein
AT8	Hyperphosphorylated tau at epitopes detected by AT8 antibody
SH3	Src Homology 3 domain
IHC	Immunohistochemistry
IBA1	Ionized calcium-binding adapter molecule 1 (microglial marker)
GFAP	Glial Fibrillary Acidic Protein (astrocytic marker)
rAAV	Recombinant adeno-associated viral vector
GC/mL	Genome copies per milliliter
SCR	Scramble (control vector)
miR30	MicroRNA-30
CMV	Cytomegalovirus promoter
PBS	Phosphate-buffered saline
EEG	Electroencephalography
PTZ	Pentylenetetrazole
TBI	Traumatic brain injury
GPx-1	Glutathione Peroxidase-1
nNOS	Neuronal Nitric Oxide Synthase
PKCδ	Protein Kinase C delta
Y18	Tyrosine 18 (of tau)
eGFP	Enhanced Green Fluorescent Protein
CNS	Central Nervous System
ARRIVE	Animal Research: Reporting of In Vivo Experiments
IACUC	Institutional Animal Care and Use Committee
NIH	National Institutes of Health
NINDS	National Institute of Neurological Disorders and Stroke
