# Supplementary material for: The Effects of Neuronal Fyn Knockdown in the Hippocampus in the Rat Kainate Model of Temporal Lobe Epilepsy
Source: Cells. 2025 May 19;14(10):743. doi: 10.3390/cells14100743 (PMC12110385; doi:10.3390/cells14100743)
Supplement: Supplementary file 1 [file cells-14-00743-s001.zip › cells-3607542-supplementary.pdf]

1. Supplementary Tables

Supplementary table 1. Details of antibodies used for Western blotting

Antibody	Host	Manufacturer	Catalog No.	Dilution
pNR2B (Y1472)	Rabbit	PhosphoSolutions	p1516-1472	1:500
NR2B	Mouse	Santa-Cruz Biotechnology	Sc-365597	1:1000
nNOS	Sheep	PC Emson	RRID: AB-2314957	1:1000
PSD95	Rabbit	Invitrogen	51-6900	1:1000
Fyn	Rabbit	Sigma-Aldrich	HPA023887	1:1000
pSFK (Y416)	Rabbit	Cell Signaling	2101S	1:500
pTau (AT8)	Mouse	Invitrogen	MN1020	1:500
Tau (DA9)	Mouse	Peter Davies	RRID: AB_2716723	1:1000
Src	Mouse	Invitrogen	AHO1152	1:1000
IBA1	Goat	Abcam	Ab5076	1:1000
GFAP	Mouse	Sigma-Aldrich	AB5804	1:1000
NeuN	Rabbit	Sigma-Aldrich	MAB377	1:2000
Beta Actin	Mouse	Sigma-Aldrich	A5316	1:10000
IRDye® 800CW or 680LT Donkey IgG (H + L)	Mouse, Rabbit, Sheep, Goat	Li-Cor Biosciences	926-32213	1:5000

2. Supplementary Figures

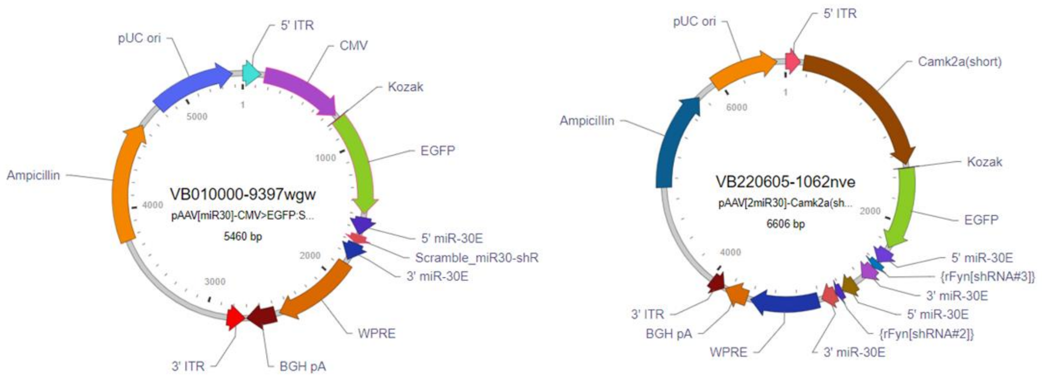

Figure S1. Vector maps of the recombinant AAV vectors used in the study.

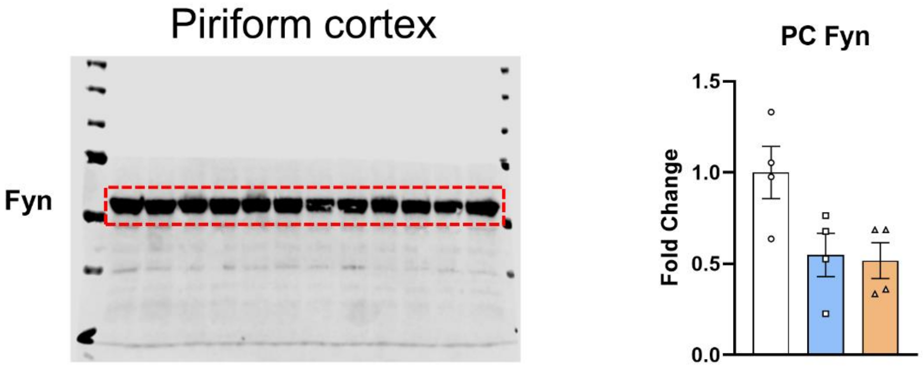

Figure S2. Fyn expression in the piriform cortex. Western blot analysis of whole piriform cortex lysate probed for Fyn expression revealed no significant differences in the expression of Fyn as seen in the hippocampus, indicating targeted hippocampal *fyn* knockdown. Ordinary one-way ANOVA (Tukey's multiple comparisons test), n= 4 per group.

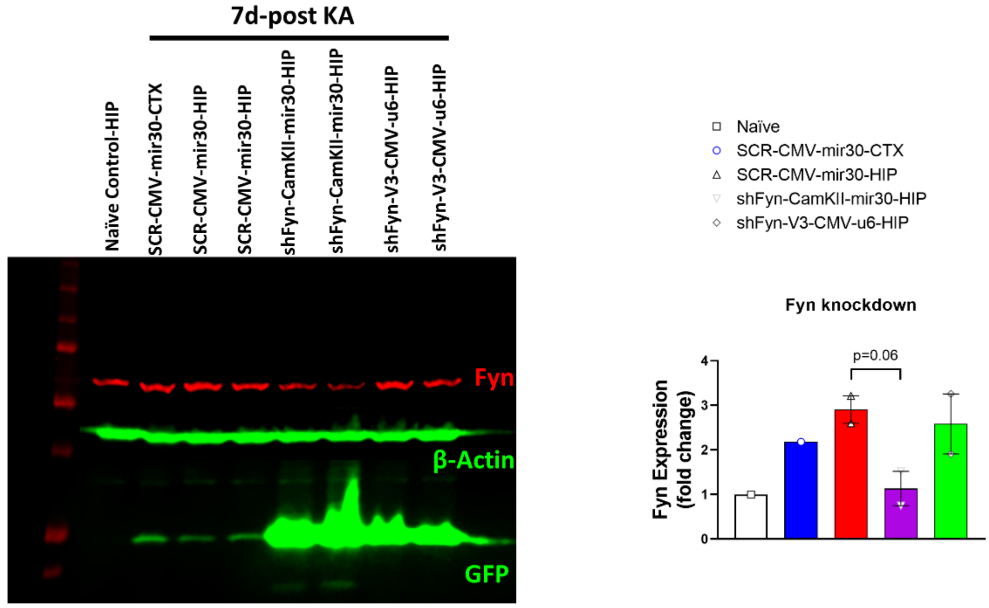

Figure S3. Preliminary data for testing AAV vector constructs. Robust *fyn* knockdown was observed in the shFyn-CaMKII-mir30 constructs, which were used in the present study for *fyn* knockdown. Unpaired t-test.

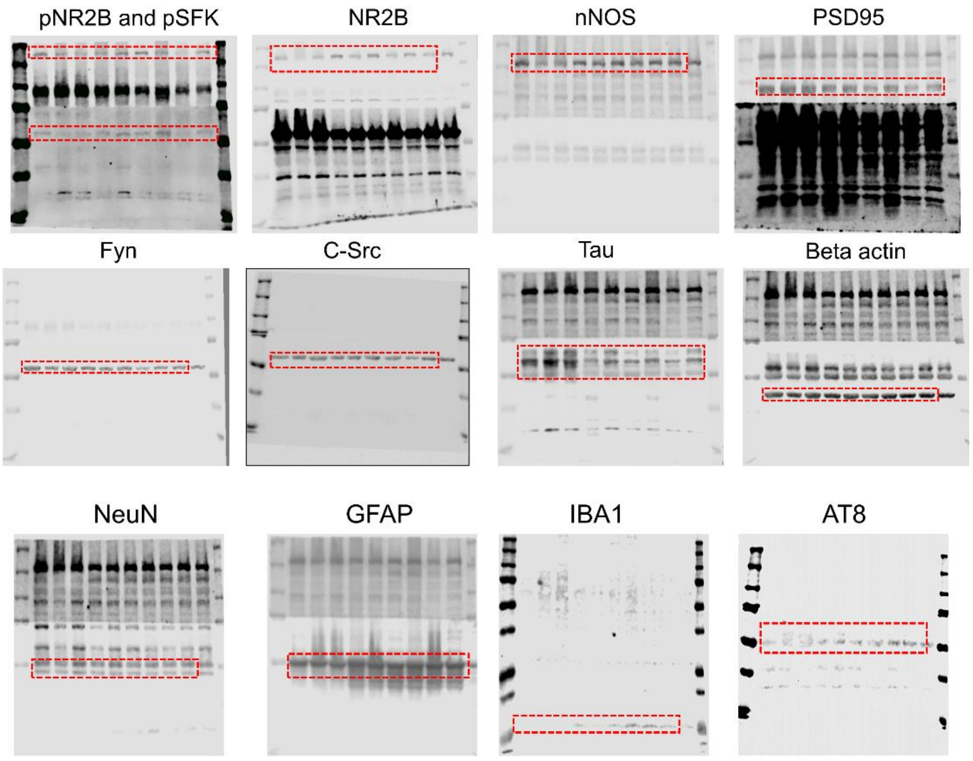

Figure S4. Whole membrane scans of the Western blots of Figures 3 and 5.

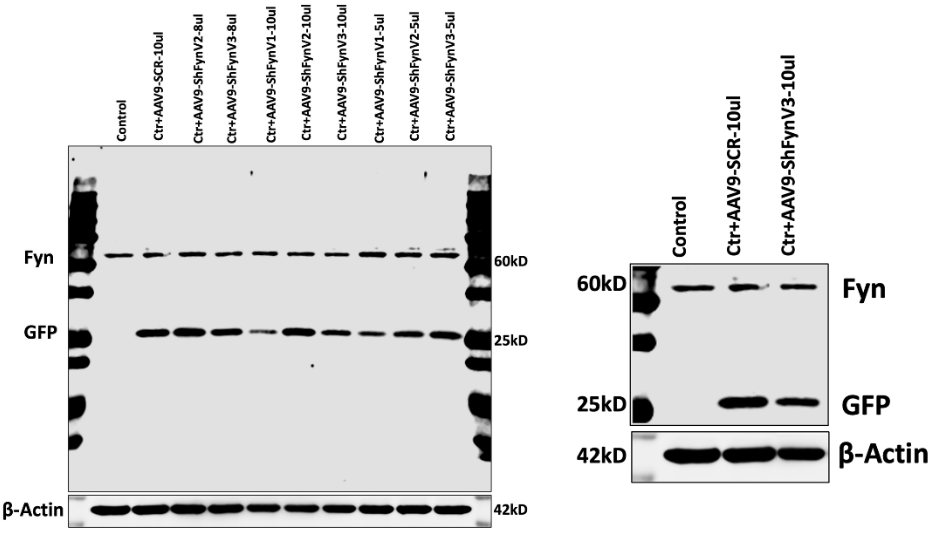

Figure S5. Preliminary in vitro studies in primary neuro-glial cultures showed no reduction in Fyn expression in any of the shRNA-treated groups.

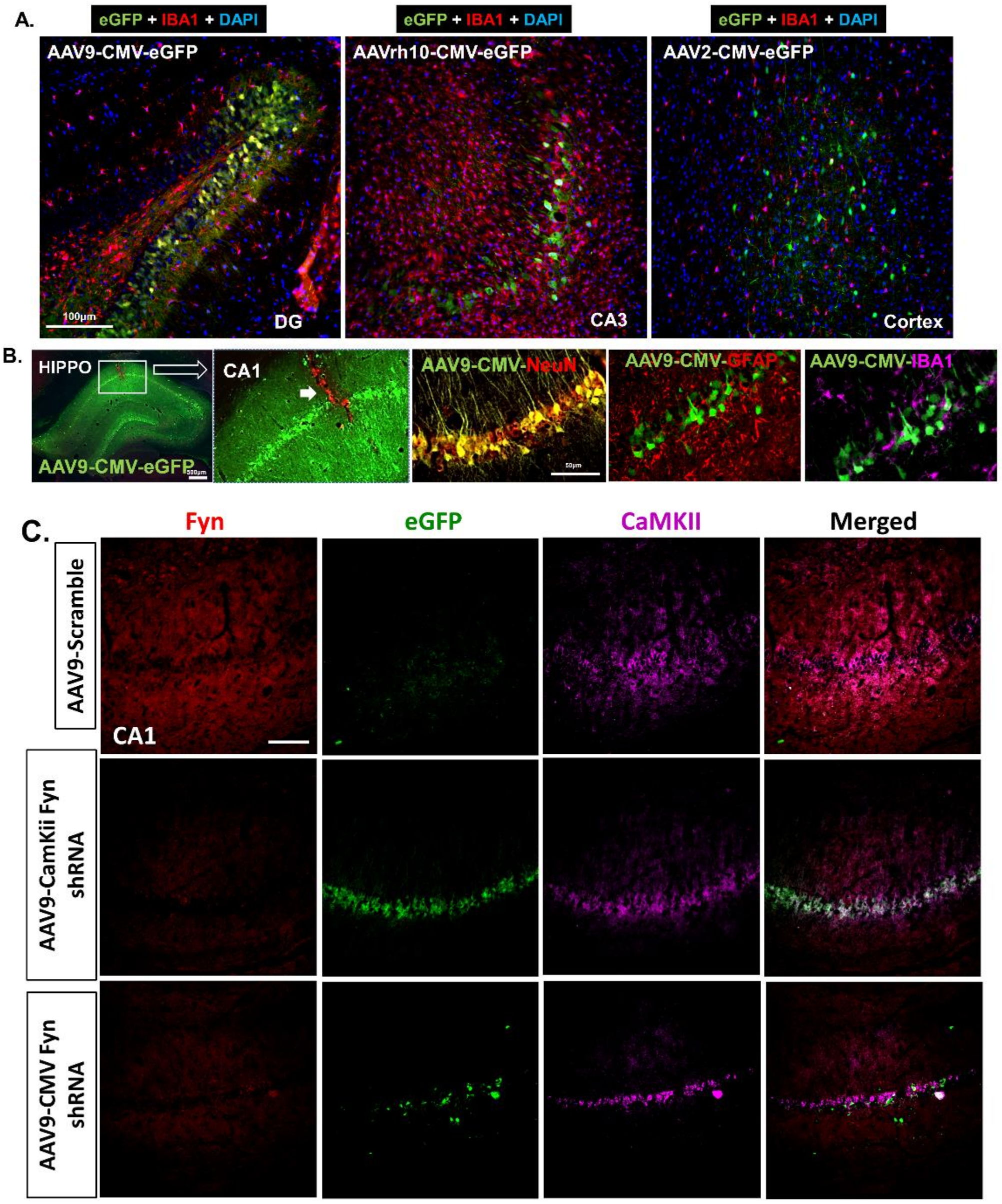

Figure S6. (A) Preliminary studies for AAV serotype testing to select effective serotypes for neuronal shRNA transduction. (B) AAV9 serotype yielded the most effective transduction of eGFP expression in hippocampal pyramidal neurons without any expression in glial cells. (C) Discrete expression of eGFP in the pyramidal neurons that also expressed CaMKII.
